# Prevalence of Cervical-Vaginal Infections in the Pap-Smear Samples in Iran

**DOI:** 10.5539/gjhs.v6n1p201

**Published:** 2013-12-19

**Authors:** Cheraghi Maria, Rahimi Zahra, Parsa Sara

**Affiliations:** 1 Social Determinant of Heath Research Center, Department of Public Heath, Health School, Ahvaz Jundishapur University of Medical Sciences, Ahvaz, Iran; 2 Department of Epidemiology, Health School Tehran University of Medical Sciences, Tehran, Iran; 3 Health Center of Dashte-Azadegan, Ahvaz Jundishapur University of Medical Sciences, Dashte-Azadegan, Iran

**Keywords:** cervical infections, vaginal infections, pap-smear, prevalence, Iran

## Abstract

Cervical-vaginal infection is one of the most common problems in clinical medicine. We aimed to determine the prevalence of cervical-vaginal infections in pap-smear samples from women in urban and rural areas. Method: It was a cross - sectional study which had done on 1448 non-pregnant women those had attended 12 health centers in the Dashte- Azadegan city during 2007-2011, Iran. After explained the aim of study, all subjects had signed informed consent, questionnaires regarding demographic and reproductive characteristics, and contraceptive methods used were completed by researcher. Also, pap-smear samples were prepared by a trained obstetrician and sent it to the pathology laboratory. All data were analyzed using SPSS (version 19). Descriptive and analytical statistics (chi – square test) were also applied. Results: The result showed that 55.9% and 44.1% of subjects were respectively in urban and rural areas. The mean age of women was 28±8.075. Pap smear results had shown that 8.8% of samples were infected with one of microorganisms such as Chlamydia, Candida, Cardnerella, and Trichomonas. A significant association was seen between contraceptive methods, education levels and place of residence with cervical-vaginal infections. Conclusion: The most prevalent pathogens by descending order were: Candida, Trichomonas and Gardenerella. The prevalence of cervical-vaginal infections was consistent with the results of many studies but it was different with the results of some studies. This could be due to the special conditions of social, economic and cultural of each area.

## 1. Introduction

Cervical-vaginal infections are one of the most common problems in clinical medicine. Gardnerella vaginalis, Candidiasis and Trichomoniasis are responsible for 90% of the cases of diseases infectious origin (Adad et al., 2001). Trichomonas vaginalis is a flagellate protozoan considered to be sexually transmittable, and can create an anaerobic environment that favors bacterial vaginosis (BV) such as Gardnerella vaginalis (Adad et al., 2001; Heller et al., 2006). The organism mlike Chlamydia trachomatis may present as vaginal discharge whereas Candida albicans and T. vaginalis have an established role in vaginitis (Sehgal & Nalini, 1990). Vulvovaginal candidiasis is the second cause of vaginitis, but it made be caused by several candida species, principally C. albicans. Is no true that C. albicans is resistant to antifungal drugs. The resistance rate to antifungals is variable and depends on the population, of type of antifungal drug, if azole or polyene (Martins et al., 2012). In general, cervical-vaginal infections are associated with significant morbidity, complications, and costly treatment (Heller et al., 2008). The cytological diagnosis (Pap-smear) was used for screening of cervical cancer, In addition, G. vaginalis, T. vaginalis and C. albicans infections can also be easily diagnosed from cervical cytology, by identification either of the organism or of characteristic cytological cellular changes (Konje et al., 1991, Escoffery et al., 2001). Since the pap-smear exam is easy to perform, well tolerated, and relatively inexpensive, the large number of women was examined by Pap –smear annually (Nokiani et al., 2008). Thus, this technique can become an important tool and the easiest method for detection of cervical-vaginal infections, especially, in developing countries (West et al., 1988). Various studies conducted to determine the frequency of the most common agents for cervical-vaginal infections have shown different results. A study in Nigeria indicated the prevalence of 9.76% for G. vaginalis, 2.52% for T.vaginalis and 2.20% for candidiasis (Konje et al., 1991). Another study reported the rate of 3.3% for Trichomoniasis (Adeoye & Akande, 2007). Even prevalence of 33% is obtained about Gardnerella (West et al., 1988). In Iran, the prevalence rate of G. vaginalis, T.vaginalis and candidiasis was 9.4%, 7.3%, and 15% respectively (Parhizgar & Moshafaa, 2002).

Since maintenance and promotion of women's health has a special value in health care, it is necessary to have the accurate information about cervical-vaginal infections to design and implement health plans. Considering few studies on the cervical-vaginal infections in pap-smear samples in Iran, this study aims to determine prevalence of cervical-vaginal infections according to age, the level of education, place of residence, the number of pregnancies, parity and abortion, and contraceptive methods.

## 2. Methods

It was a cross - sectional study which had done on 1448 non-pregnant women those had attended 12 health centers in the Dashte- Azadegan city in Khuzestan province, Iran, during 2007-2011.

We had invited 1448 non-pregnant women to participate this study, all of them had attended 12 health centers (including 8 rural health centers and 4 urban health centers) and they had written informed consent. We had confirmation Ethics Committee from Ahvaz Jundishapour university of Medical Sciences AJMSU. Inclusion criteria were married women, age to be less than 70 years old and had to have lived in Dasht-e- Azadegan city, and all Virgins, pregnant and menstruating women were excluded from this study.

### 2.1 The Pap-Smear Sampling Method

Papanicolaou test (also called Pap smear, Pap test, cervical smear, or smear test) is a screening test used to detect potentially pre-cancerous and cancerous processes in the endocervical canal (transformation zone) of the female reproductive system. Unusual findings are often followed up by more sensitive diagnostic procedures, and, if warranted, interventions that aim to prevent progression to cervical cancer. The test was invented and named after the prominent Greek doctor Georgios Papanikolaou. In taking a Pap smear, a speculum is used to open the vaginal canal and allow the collection of cells from the outer opening of the cervix of the uterus and the endocervix. The cells are examined under a microscope to look for abnormalities

### 2.2 Data Analysis

All data were analysed using SPSS (version 19). To analyze, descriptive statistics such as frequency tables, mean, and standard deviation was used. As well as chi-square test was applied to find the association between two variables. The significance level of 0.05 was considered for statistical analysis.

## 3. Results

This study had shown that 1448 women enrolled among those attended at health centers in rural (44.1%) or urban (55.9%) areas. The mean age of women was 28±8.075. The mean number of gravidity, parity, and abortion was respectively 4±2.92, 3.6±3.2, and 0.4±0.89. Most of participants had up to 30 years of age, were illiterate or had only elementary education, living in urban areas and had of 4-5 years of work time (Table 1).

**Table 1:** General characteristics of study participants (n=1448)

Variable		Number	Percent
**Level of education**	Illiterate	417	28.8
	Elementary	443	30.6
	<High school	253	17.5
	High school or equal	272	18.8
	University degree	63	4.4
**Age**	<20	147	10.2
	21-30	631	43.6
	31-40	466	32.2
	41-50	163	11.3
	>50	41	2.8
**Place of residence**	Urban	809	55.9
	Rural	839	44.1

Pap smear results showed that approximately 8% of samples were infected with one of microorganisms such as *Chlamydia*, *Candid*,*Gardnerella*, and *Trichomonas*. The highest rate of cervical-vaginal infection was due to Candida so that 6% of infections were included. The most prevalence of cervical-vaginal infections was in the age group of 21-30 years and lowest rate was observed in the age group above 50years. A significant association was not seen between age groups and cervical-vaginal infections (Table 2).

**Table 2:** Distribution of participants according to fertility characteristics (n=1436)

Variable		Number	Percent
**Gravity**	No	54	3.8
	1-3	941	65.6
	4-6	345	24
	>6	96	6.6
**Parity**	No	65	4.5
	1-3	988	68.8
	4-6	315	21.9
	>6	68	4.8
**Abortion**	No	1193	83.1
	1-3	236	16.4
	>3	7	0.5

It was reported use of the following contraceptive methods: Low-dose estrogen (LD), Combined oral contraceptives - also called of "the pill" (TP), Long-term hormonal contraceptive applied intramuscularly (DMPA), Intrauterine device (IUD), Condom, Tubal ligation (TL), Natural, None (not considered necessary), Menopause and other. Among the contraceptives methods that were cited, LD was the most common, being used by 34.3% of women participating in the study, followed by the condom used by 15.5% of them. A significant proportion of women (11.7%) reported not using any contraceptive method, by not find it necessary (Table 3). Also a significant association was between contraceptive methods and cervical-vaginal infections (p<0.001).

**Table 3:** Distribution of participants according to the current method of contraception (n=1443)

The methods of contraception	Number	Percent
**LD**	495	34.3
**TP**	102	7.1
**DMPA**	63	4.4
**IUD**	47	3.2
**CONDOM**	224	15.5
**TL**	10	7.6
**Natural**	128	8.9
**None**	169	11.7
**Menopause**	13	0.9
**Other (MP, CYCLO, and etc.)**	92	6.4

As can be seen in Table 4, high prevalence of cervical-vaginal infection was seen respectively in illiterate women and women with the primary education. Also a significant association was between education levels and cervical-vaginal infections (p=0.018). The prevalence of cervical-vaginal infections was more in rural women than urban women and there was a significant association between residence and cervical-vaginal infections (p<0.001).

**Table 4:** Distribution of cases of cervical-vaginal infections based on demographic characteristics and contraceptive methods used

	**The type cervical infection**							
Variables	None pathogen	*Chlamydia*	*Condida*	*Gardenerella*	*Trichomonas*	Other*	Total	*P value*
**Level of education**								*0.018*
Illiterate	187 (44.8%)	1 (2.0%)	3 (0.7%)	187 (44.8%)	6 (1.4%)	185 (44.4%)	417	
Elementary	192 (43.3%)	1 (0.2%)	4 (0.9%)	192 (43.3%)	4 (0.9%)	212 (47.9%)	443	
Middle school	85 (33.6%)	0 (0.0%)	3 (1.2%)	85 (33.6%)	2 (0.8%)	147 (58.1%)	253	
High school or equal	100 (36.8%)	1 (0.4%)	1 (0.4%)	100 (36.8%)	2 (0.7%)	160 (58.8%)	272	
University degree	100 (36.8%)	0 (0.0%)	0 (0.0%)	21 (33.3%)	0 (0.0%)	40 (63.5%)	63	
**Age**								*0.31*
<20	57 (38.8%)	0 (0.0%)	14 (9.5%)	1 (0.7%)	2 (1.4%)	73 (49.6%)	147	
21-30	257 (40.7%)	0 (0.0%)	40 (6.3%)	7 (1.1%)	4 (0.6%)	323 (51.2%)	631	
31-40	187 (40.1%)	2 (0.4%)	24 (5.2%)	2 (0.4%)	4 (0.9%)	247 (53.0%)	466	
41-50	65(39.9%)	1 (0.6%)	10 (6.1%)	1 (0.6%)	4 (2.5%)	82 (50.3%)	163	
>50	19 (46.3%)	0 (0.0%)	3 (7.3%)	0 (0.0%)	0 (0.0%)	19 (46.3%)	41	
**Place of residence**								*<0.001*
Urban	271 (33.5%)	2 (0.2%)	34 (4.2%)	8 (1.0%)	11 (1.4%)	483 (59.7%)	809	
Rural	314 (49.1%)	1 (0.2%)	57 (8.9%)	3 (0.5%)	3 (0.5%)	261 (40.8%)	639	
**Contraceptive methods**								*<0.001*
LD	212 (42.8%)	2 (0.4%)	40 (8.1%)	7 (1.4%)	7 (1.4%)	239 (48.3%)	495	
TP	45 (44.1%)	0 (0.0%)	7 (6.9%)	1 (1.0%)	3 (2.9%)	46 (45.1%)	102	
DMPA	34 (54.0%)	0 (0.0%)	1 (1.6%)	0 (0.0%)	0 (0.0%)	28 (44.4%)	63	
IUD	10 (21.3%)	0 (0.0%)	2 (4.3%)	2 (4.3%)	0 (0.0%)	33 (70.1%)	47	
CONDOM	89 (39.7%)	0 (0.0%)	11 (4.9%)	0 (0.0%)	2 (0.9%)	122 (54.5%)	224	
TL	42 (38.2%)	0 (0.0%)	8 (7.3%)	0 (0.0%)	2 (1.8%)	58 (52.7%)	110	
Natural	50 (39.1%)	0 (0.0%)	8 (6.3%)	1 (0.8%)	0 (0.0%)	69 (53.8%)	128	
None	58 (34.3%)	1 (0.6%)	11 (6.5%)	0 (0.0%)	0 (0.0%)	99 (58.6%)	169	
Menopause	4 (30.8%)	0 (0.0%)	0 (0.0%)	0 (0.0%)	0 (0.0%)	9 (69.2%)	13	
*Other*	*36 (40.0%)*	*0 (0.0%)*	*3 (3.3%)*	*0 (0.0%)*	*0 (0.0%)*	*53 (56.7%)*	*92*	

The results showed that there was no significant association between cervical-vaginal infections with the number of gravidity, parity, and abortion (p>0.05).

## 4. Discussion

This study has shown that most cervical-vaginal infections were attributable to Candida and then Trichomonas and Gardnerella. Other studies indicated the prevalence of Candida was 25.2%, the rate of Trichomonas was 1.37%, and 25% for Gardnerella (Namazi et al., 2005; Molana & Ghasi-Saeidi, 2000; Sadeghi & Naeini, 2001). These differences are due to different study populations, various climate, socio-economic and cultural conditions, different sensitivity of diagnostic tests, applying different diagnostic methods (Parhizgar & Moshafaa, 2002; Namazi et al., 2005). Also the studies demonstrated that T. vaginalis is a STD pathogen and related to low socioeconomic levels (Adad et al., 2001).

About contraceptive methods used, the highest rates of Candida infections were seen in women who were using LD. The results of other studies confirm this finding (Namazi et al., 2005) because combined oral contraceptive play an important role in the incidence of vaginal yeast infections (Parhizgar & Moshafaa, 2002). The highest prevalence of Gardnerella infections was related to LD and IUD users, which consistent with the result of other studies (Parhizgar & Moshafaa, 2002). In current study, similar to another study (Habibipour & Bayat, 2009), the majority of participants were using LD as the contraceptive method while another study in Tabriz showed that most women used IUD for contraception (Namazi et al., 2005).

Our study showed that there was no significant association between cervical-vaginal infections with the gravidity, parity and abortion. The study in Tabriz also reported no association between the number of abortion and these infections whereas some surveys reported an association between parity, Candida and Trichomonasis (Namazi et al., 2005, Nourian et al., 2011).

The findings indicate that women aged up to 30 years had the highest prevalence rates of cervical-vaginal infection by Candida and Gardenerella, while the highest rates of prevalence of cervical-vaginal infection by Chlamydia and Trichomonas were observed in the women aged 41 to 50 years. None of the other pathogens, except Candida was found in the women over 50 years of age old. This is consistent with the result of many studies (Adad et al., 2001; Namazi et al., 2005; Gouya and Nabaei, 2005). This is probably due to high sexual activity and, possibly greater tendency of change of sexual partners in the this age group (Parhizgar and Moshafaa, 2002).In present study a significant association was not seen between age groups and cervical-vaginal infections but there was an association between education levels, place of residence with cervical-vaginal infections.

## 5. Conclusion

The prevalence of cervical-vaginal infections was consistent with the results of many studies but it was different with the results of some studies. This could be due to the special conditions of social, economic and cultural of each region. Our finding has shown that a high prevalence of cervical-vaginal infections in women with aged up to 30 years, so it is necessary to have the accurate information about cervical-vaginal infections to design and implement health plans.
